# Mr. Lizars on Division of Submaxillary Nerve

**Published:** 1830-07-01

**Authors:** 


### XLII.

### DIVISION OF THE SUBMAXILLARY AND OTHER NERVES.

*To the Editor of the Medico-Chirurgical Review, London.*

*Sir*,—I observe in your fasciculus for February last, the description of an operation performed by Dr. Warren, for excision of the submaxillary nerve, extracted from the Boston Medical and Surgical Journal. This mode of operating appears truly formidable, and is probably that which the late Dr. Haighton had in view, when he pronounced the division of this nerve impracticable.

A much simpler, safer, and easier mode of accomplishing the division of this nerve, where it enters the canal of the inferior maxillary bone, is, to make an incision, with a scalpel, from within the mouth to the extent of an inch, through the mucous membrane and cellular tissue connecting the pterygoideus internus muscle, to the ramus of the bone, parallel and close to the inner or mesial surface of the coronoid process immediately behind the dens sapientiæ; then to take a round-shaped gum lancet and carry it backwards in a line continuous with the crowns of the molar teeth, having the cutting edge at right angles to the bone, and divide the nerve on the bone. The pain experienced on the division of the nerve, at once, indicates that the proper organ has been cut. As the internal maxillary artery ascends to the bulbous process of the superior maxillary bone, it cannot be wounded, excepting through ignorance or carelessness; but, even if it were, a piece of dry sponge might be easily inserted to stem the hæmorrhage. The gustatory branch of the nerve could scarcely be injured. The dental artery must be wounded, but this is so small as to be of no moment, and, if morbidly enlarged, dry sponge would compress it. I have now performed this operation on four patients for neuralgia of the mental nerve, with perfect success, having previously attended to the chylopoietic viscera, and then tried the various antispasmodics and subcarbonate of iron; also the different counter-irritants, even the moxa, and lastly, the division of the nerve as it emerges at the mental foramen. Or, according to your own showing—"after the local symptoms from morbid associations or change of structure, had continued after the constitutional derangement from which they originally emanated, had been rectified—and the consequence had survived the cause." My first case was published in the Edinburgh Medical and Surgical Journal for October, 1821.

Thus there would appear to be a material difference between the division of the trunk of a nerve, where it is protected from the vicissitudes of atmospherical influence by muscular and other soft coverings, and the division of the same nerve, where it is exposed to the alternations of the weather, as far as relates to the permanent salutary result. It is well known, that in neuroma supervening to amputation, the excision of the tumor or tumors, proves a more permanent or radical cure than a secondary amputation, also in neuralgia following the same operation, excision of the nerves does the same, and evidently in consequence of excision preventing the interesting junction of the nerves, as well as the production of the numerous delicate filaments supplying the cicatrix of the stump. This has been satisfactorily described by Larrey in his late valuable work 'Clinique Chirurgicale,' and also by Descot, in his interesting 'Dissertation sur les Affections Locales des Nerfs.' For the same reasons, the excision of a portion of a nerve must be a more effectual cure than simple division of the same.

If this view of the operative department of the pathology of nerves be found to be correct, it would follow, that the division of the infra-orbitary nerve, where it enters the osseous canal in the floor of the orbit, would prove more availing, than its division at the infra-orbitary foramen on the cheek. This might be easily accomplished as follows:—let an incision about an inch long, of a curvilinear figure, to correspond with the circular shape of the orbit, be made at the outer canthus of the eye, the centre of which shall be opposite the outer commissure or angle of the eye-lids, or rather the superior margin of the zygoma. This incision is to be deepened by cutting close to the osseous wall of the orbit, until the instrument reach the spheno-maxillary fissure, when it is to be laid aside, and a round shaped gum lancet inserted in the wound with its cutting edge at right angles to the floor of the orbit, and the nerve divided as it runs in the osseous channel. In some, this is an open, while in others, it is a shut or entire canal; but, in all, the parietes are so delicate as to be easily cut across. A portion of the infra-orbitary nerve, at its emergence from the infra-orbitary foramen, could not be removed, in consequence of its division into so many minute filaments: neither could this be accomplished within the orbit.

The supra-orbitary or frontal nerve may be also divided within the orbit, nearly an inch from the superciliary ridge, by first ascertaining the superciliary foramen or notch, which is done by drawing a perpendicular line from the second bicuspis at right angles to the area of the crowns of the teeth; secondly, by making an incision about the fourth of an inch parallel and close to the superciliary ridge at the foramen, through the integuments, orbicularis palpebrarum muscle, and ligament of the superior tarsus; thirdly, substituting for the straight bistoury or scalpel, a probe-pointed bistoury, which is to be inserted deep in the orbit close to the bone, and with which the nerve is to be divided by cutting upwards on the bone, in the direction from the inner to the outer canthus, carefully guarding against injuring the superior oblique muscle on its inner or mesial aspect. A portion of this nerve may be excised either within or without the orbit: within, as just directed, combined with searching for the nerve at the superciliary foramen, and after its division seizing hold of it with the dissecting forceps and removing the insulated or detached part. As it sends off minute filaments on its emergence from the orbit, the removal of a portion without the cavity, would not hold out a prospect of so permanent a cure.

JOHN LIZARS.

*Edinburgh*, 34, *North Place*.
*24th April*, 1830.

